# Identification and Genomic Insights into the Biological Control and Growth-Promoting Mechanism of *Bacillus velezensis* L11-7, a Potential Biocontrol Agent of Passion Fruit Stem Basal Rot

**DOI:** 10.3390/microorganisms13092084

**Published:** 2025-09-07

**Authors:** Ming Jin, Yuanfeng Tang, Rui Yang, Quan Zeng, Mingxiao Duan, Jieqiu Li, Jiaorong Meng

**Affiliations:** 1 Guangxi Key Laboratory of Sugarcane Biology, College of Agriculture, Guangxi University, Nanning 530004, China; 2 College of Life Science and Technology, Guangxi University, Nanning 530004, China; 3 Agri-Animal Industrial Development Institute, Guangxi University, Nanning 530004, China

**Keywords:** passion fruit, *Fusarium solani*, *Bacillus velezensis*, biological control

## Abstract

Basal stem rot caused by *Fusarium solani* is among the most destructive soil-borne diseases affecting passion fruit (*Passiflora* spp.). While biological control employing antagonistic microorganisms offers a promising plant protection strategy, reports on antagonists specifically targeting passion fruit basal stem rot remain limited. Here, a screen for *F. solani* antagonists led to the identification of *Bacillus velezensis* strain L11-7, whose whole genome was subsequently sequenced. Pot experiments demonstrated that strain L11-7 significantly reduced the severity of stem basal rot, achieving control efficiencies of 92.85%, and exhibited broad antagonistic properties against other plant pathogenic fungi. L11-7 possesses cellulase, glucanase, and protease activities, alongside capabilities for nitrogen and phosphorus production. L11-7 was identified as *B. velezensis* through morphological analysis, 16S rRNA, *gyrB*, and *rpoB* gene sequencing, and whole-genome analysis. Its genome features a single circular 3.97 Mb chromosome harboring 13 s metabolite biosynthetic gene clusters (e.g., fengycin, surfactin, macrolactin H, bacillaene, difficidin) and genes encoding essential cell wall hydrolases. Several genes related to plant growth promotion, including those involved in nitrogen fixation and IAA production, are also present. These results indicate that *B. velezensis* L11-7 is a prospective biocontrol agent against passion fruit basal stem rot and has plant growth-promoting properties.

## 1. Introduction

Passion fruit (*Passiflora* spp.) is a member of the Passifloraceae family, which features over 500 species [1,2]. It is widely cultivated in tropical and subtropical regions globally, notably in South America, the Caribbean, South Florida, South Africa, and Asia [3,4]. There are also extensive passion fruit cultivation areas in southern and southwestern China, including Guangxi, Guangdong, Hainan, and Taiwan [5]. Valued for its rich content of dietary fiber, minerals, vitamins, pectin, antioxidants, and flavonoids, passion fruit holds significant economic importance and has been widely developed in the food, pharmaceutical, horticulture, and byproduct processing industries [1,3,6].

However, passion fruit plants are highly susceptible to various diseases throughout their development, including those caused by nematodes, viruses, bacteria, and fungi [7,8,9,10,11]. Among these, one of the most prevalent and devastating is passion fruit stem basal rot [5,12]. Symptoms typically manifest as dark brown, dry, and cracked lesions on the stem base, 5–15 cm above ground. These lesions progressively expand and encircle the stem, leading to cortical decay, sloughing, and the browning of vascular tissues, eventually causing separation from the xylem. In severe cases, branches wilt, ultimately resulting in plant death [7,13]. In southern China’s production areas, the incidence of basal stem rot has reached 30–40% in severely affected orchards [12], making it the primary threat to the sustainable global development of the passion fruit industry [11,12]. Common approaches to controlling passion fruit stem basal rot include developing resistant cultivars or rootstock varieties for grafting, implementing crop rotation, using chemical pesticides, and employing T-shaped trellises to improve airflow and ventilation [14,15,16]. However, there are only a few commercially available resistant cultivars, making cost-effective chemical control a preferred method. Nevertheless, the overuse of fungicides leads to fungicide resistance, food safety risks, and ecological concerns [17].

Biological control, employing beneficial microorganisms, offers an environmentally friendly alternative with significant potential for sustainable crop management [18]. Widely applied biocontrol microorganisms primarily include *Bacillus* spp., *Pseudomonas* spp., *Streptomyces* spp., and *Trichoderma* spp. [19]. *Bacillus* spp., in particular, produce numerous antimicrobial compounds (e.g., hydrolases and secondary metabolites), and can be effective at inhibiting plant pathogens [20]. Additionally, *Bacillus* can promote plant growth and induce systemic resistance, and also exhibits strong adaptability and resistance to a range of environmental stresses [21]. Furthermore, *Bacillus* can reproduce rapidly under artificial cultivation conditions, allowing for high-efficiency, large-scale production of antibacterial substances through fermentation [22]. Consequently, *Bacillus* is the most widely used genus in plant disease control, with species such as *B. subtilis*, *B. velezensis*, and *B. amyloliquefaciens* successfully employed as biocontrol agents in agriculture [19].

Reports of *Bacillus* species being used for the biological control of passion fruit diseases are limited. For instance, the endophytic *Bacillus subtilis* strain GUCC4, isolated from passion fruit, significantly reduced fungal growth and spore germination in *Nigrospora sphaerica*, the pathogen causing passion fruit leaf blight [23]. Additionally, two *B. subtilis* strains, 151B1 and YBC, isolated from native rhizosphere soil, showed antagonistic activity against *Fusarium solani* PF7 and reduced the severity of *Fusarium* wilt on the leaves of passion fruits [14]. Despite these examples, relatively few beneficial microorganisms suitable for use in the biological control of passion fruit diseases have been identified to date.

Various pathogens are known to contribute to passion fruit basal stem rot, including *F. solani*, *Lasiodiplodia theobromae*, and *F. oxysporum*, with *F. solani* being the most prevalent [7,12,13,14,24,25]. In this study, we screened an antagonistic *Bacillus* strain L11-7, which is effective against *F. solani* and was identified as *Bacillus velezensis*. Through comprehensive morphological analysis, multi-gene phylogenetic analysis, and genome annotation, L11-7 was identified as *B. velezensis*. We further investigated the antagonistic potential, biocontrol efficacy, and plant growth-promoting (PGP) properties of L11-7. Finally, whole-genome sequencing (WGS) of strain L11-7 was performed to identify genes or gene clusters potentially related to secondary metabolite biosynthesis and plant growth promotion. Our findings will facilitate the development of biological strategies for the control of stem rot disease caused by *F. solani* in passion fruits.

## 2. Materials and Methods

### 2.1. Strains and Plant Materials

The *Fusarium solani* strain K3-5 was isolated from the diseased stem bases (exhibiting dark brown and cracked lesions) of the passion fruit cultivar Tainong No.1 in 2022 in Wuming County, Guangxi Zhuang Autonomous Region, China. The other phytopathogenic fungal strains were isolated from diseased samples collected by our team between 2019 and 2023. All the fungal strains were confirmed morphologically and molecularly and were preserved at −80 °C. The 43 *Bacillus* strains used in this study were isolated from the stem or branch of mulberry or the rhizosphere soil of healthy plants. All the strains were stored at –80 °C. The *Bacillus* and phytopathogenic fungal strains used in this study are listed in Table S1. For use in experiments, the phytopathogenic fungi were grown on Potato Dextrose Agar (PDA) (g/L: dextrose 20, potato extract 4, agar 15) plates at 28 °C for 8 days in the dark, while the *Bacillus* strains were cultured on solid Luria–Bertani (LB) medium (g/L: tryptone 10, yeast extract 0.5, and NaCl 10) for 2 days.

Seedlings of passion fruit Tainong No.1, susceptible to *F. solani*, were provided by Huaxiang Seedling Co., Ltd., Naning, the Guangxi Zhuang Autonomous Region, China.

### 2.2. Screening of Antagonistic Strains Against F. solani In Vitro

The antagonistic activities of the *Bacillus* strains against *F. solani* were evaluated using the modified dual-culture plate assay [26]. For preliminary screening, a mycelial plug (6 mm in diameter) of *F. solani* was placed at the center of a PDA plate, and four different tested *Bacillus* strains were each spot-inoculated 2 cm from the plug. Plates inoculated only with *F. solani* were prepared as a control, with three replicates being applied for each *Bacillus* strain. The plates were incubated at 28 °C for 8 days in the dark. The presence of an inhibition zone preliminarily indicated that the *Bacillus* strain exhibited antagonistic activity against *F. solani*. For secondary screening, a mycelial plug of *F. solani* was inoculated at the center of a PDA plate as described above. The candidate antagonistic strains, namely, those that presented potential antagonistic activity in the preliminary screening, were inoculated on both sides of the mycelial plug at a distance of 2 cm. The control plate was not inoculated. Each treatment was replicated three times. The plates were incubated at 28 °C for 8 days in the dark. The inhibition effect was observed, and the percent inhibition was calculated as follows:

(1) $$Inhibition\;rate\;\left(\%\right)=\frac{Cc-Tc}{Cc}\times 100$$

where Cc and Tc are the colony diameters of the plant pathogens in the control and treatment groups, respectively. The *Bacillus* strains exhibiting an inhibition rate exceeding 60% were selected for the pot experiments.

### 2.3. Pot Experiments Assessing the Biocontrol Potential of Antagonistic Bacillus Strains

For *Bacillus* strain suspensions, each test bacterium was cultured for 2 days, after which a single colony was inoculated into 100 mL of LB liquid medium and incubated at 28 °C with shaking at 200 rpm to an optical density (OD600) of 1.0. Subsequently, a 2% (*v*/*v*) inoculum was transferred to fresh LB liquid medium and further incubated under identical conditions for 1 day to prepare bacterial fermentation broth, which was then diluted 30 times.

Healthy seedlings of Tainong No.1 of uniform size and growth conditions were selected and planted in the greenhouse at a temperature of 28 °C. At the same height on the stem of each plant, wounds were made via a single prick with a plum-blossom needle. The edge of the mycelium of *F. solani* that had been grown on PDA plates at 28 °C for 8 days in the dark was taken and placed on the wound surface. Each treatment group consisted of 15 passion fruit seedlings. For the treatment group, 5 mL of the bacterial fermentation solution was sprayed onto the surface of each seedling, and then 15 mL was sprayed at the base of the stem. Two control groups were set up, one consisting of inoculation with non-sterile PDA medium, and the other inoculation with *F*. *solani* and spraying with water. The plants were completely covered and surrounded with plastic film to ensure humidity. On day 5, the treatments were reapplied once. On day 15, the lesion size on the stem wounds was measured, and the area of the diseased parts of the plants and the control effect (%) were calculated to identify the dominant biocontrol strain for the subsequent study.

(2) $$Control\;effect\;(\%)=\frac{Cl-Tl}{Tl}\times 100$$

where Cl and Tl are the plant lesion areas of the control and treatment groups, respectively.

### 2.4. Assessment of the Antagonistic Spectrum of L11-7

To assess the antagonistic spectrum of strain L11-7, its inhibitory effect against 16 plant pathogenic fungal strains was further evaluated using the double culture method described above. Plates inoculated only with the fungal pathogen served as controls. Each treatment was replicated three times. The phytopathogenic fungi tested in this study are listed in Table S1.

### 2.5. Assay of the Biological Control and Plant Growth-Promoting Traits of L11-7 In Vitro

The hydrolase activities of strain L11-7, including protease, cellulase, xylanase, and β-1,3-glucanase, were evaluated as previously described [27,28]. The nitrogen-fixing ability of strain L11-7 was evaluated using nitrogen-free medium as described by Bolivar-Anillo et al. [29], while indole-3-acetic acid (IAA) production, potassium solubilization, and phosphate dissolution abilities were assessed according to the methods described by Pour et al. [30] and Wang et al. [5].

### 2.6. Morphological Observation of L11-7

Strain L11-7 was incubated at 28 °C for 2 days, following which single colonies were picked for Gram staining. The stained slides were observed using a microscope equipped with an oil immersion objective, and the staining results were recorded. For the SEM analysis, 10 mL of bacterial fermentation broth (OD600 = 0.7) was centrifuged at 12,000× *g* for 2 min, the resulting pellet was washed three times with 1× phosphate-buffered saline (PBS), and centrifuged. After removing the supernatant, glutaraldehyde fixative (2.5%, *v*/*v*) was added to immobilize the bacterial cells, which were then imaged using a scanning electron microscope (JSM-IT 700HR/LV, JEOL, Tokyo, Japan).

### 2.7. DNA Extraction, Polymerase Chain Reaction (PCR), and Molecular Identification of Strain L11-7

Genomic DNA was extracted from strain L11-7 using a modified boiling method [31]. Single colonies cultured on a solid LB plate for 2 days were picked and inoculated into 10 mL of LB medium, incubated at 28 °C for 14 h with shaking (200 rpm), and then centrifuged at 12,000 rpm for 5 min. After discarding the supernatant, 500 μL of sterile water was added, and the suspension was shaken to obtain a homogeneous bacterial suspension. The sample was then placed at −20 °C for 30 min, followed by boiling in a water bath for 5 min. After a final 10-min centrifugation step, the supernatant was collected and used as the genomic DNA solution.

PCR amplification of the 16S rRNA, *gyrB*, and *rpoB* genes was performed using the DNA extracted from L11-7 as a template. The PCR mixtures (50 μL) contained 25 μL of PremixTaq (ExTaq v.2.0 with loading dye), 2 μL each of forward and reverse primers, 2 μL of DNA template, and 19 μL of nuclease-free water. PCR amplification was performed on a ProFlex Base thermal cycler (Thermo Fisher Scientific, Waltham, MA, USA). The list of primers and the PCR cycling conditions are detailed in Table S2. The PCR products were purified using the HiPure Gel Pure Mini Kit (Shanghai, China), cloned and processed using the pEASY-T1 Cloning Kit (Beijing, China), and sequenced.

The obtained 16S rRNA, *gyrB*, and *rpoB* gene sequences were compared with the sequences of *Bacillus* strains in the National Center for Biotechnology Information (NCBI) database. Next, phylogenetic analysis was performed using MEGA11.0 software, where neighbor-joining trees were constructed with 1000 bootstrap replicates.

### 2.8. Genome Sequencing, Assembly, and Annotation

Whole-genome sequencing and assembly of strain L11-7 were performed by Wuhan Beina Technology Co., Ltd., Wuhan, China. The raw sequencing data generated via the Nanopore PromethION and Illumina NovaSeq 6000 platforms were assembled using Unicycler (v.0.5.0). Gene prediction for the assembled genome was carried out using Prokka (v.1.14.6) [32]. Gene functional annotation was performed using multiple databases, including KEGG, GO, and COG [33,34,35]. Secondary metabolite biosynthetic gene clusters were predicted using the antiSMASH 6.0 online platform [36]. Additionally, genes encoding carbohydrate-active enzymes were annotated and analyzed using the CAZy database. For genomic taxonomy analysis, ANI was calculated using the JSpeciesWS online platform (https://jspecies.ribohost.com/jspeciesws/) (accessed on 2 March 2025) [37]. dDDH values were determined using the Genome-to-Genome Distance Calculator (GGDC) (http://ggdc.dsmz.de/home.php) (accessed on 3 March 2025) [38].

### 2.9. Statistical Analysis

The statistical graphs were created using GraphPad Prism 10.1.2. One-way analysis of variance and least significant difference analyses were performed using IBM SPSS Statistics 22, with *p* < 0.05, *p* < 0.01, and *p* > 0.05 defined as statistically significant, extremely significant, and no significant difference, respectively.

## 3. Results

### 3.1. Antagonistic Bacillus Screening and Biocontrol Assessment for Passion Fruit Stem Base Rot

**Figure 1:**
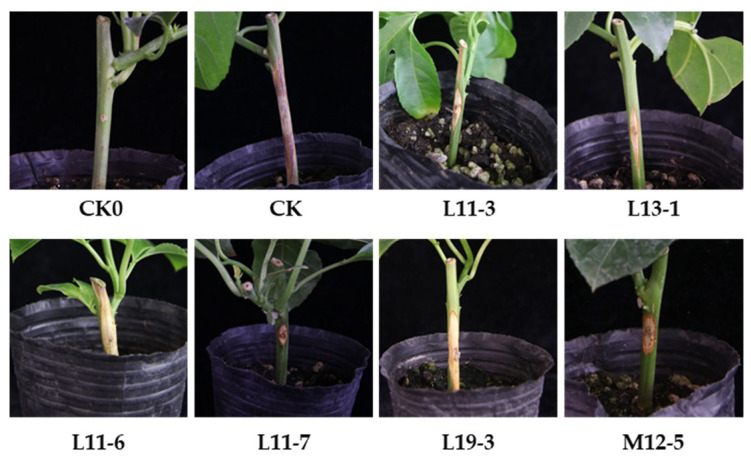
Representative disease lesions on potted plants in the biocontrol assay. Fifteen plants per group were inoculated and cultivated at 28 °C for 15 days. CK: Water spray.

A total of 43 *Bacillus* strains, collected in our previous studies, were screened for antagonistic activity against *Fusarium solani* using a dual-culture plate assay. Eighteen strains showed varying degrees of antagonism (Figure S1 and Table S3). The *Bacillus* strains (L11-3, L11-6, L11-7, L13-1, L19-3, and M12-5) exhibiting an inhibition rate exceeding 60.00% were selected for the pot experiments. Unexpectedly, strain L11-7, with the lowest inhibition rate, significantly reduced stem basal rot damage, exhibiting the highest efficacy, with a 92.85% control effect (Figure 1 and Table S4). These promising results led to the selection of L11-7 for further characterization as a biocontrol candidate.

### 3.2. L11-7 Exhibited Broad-Spectrum Antifungal Activity

**Figure 2:**
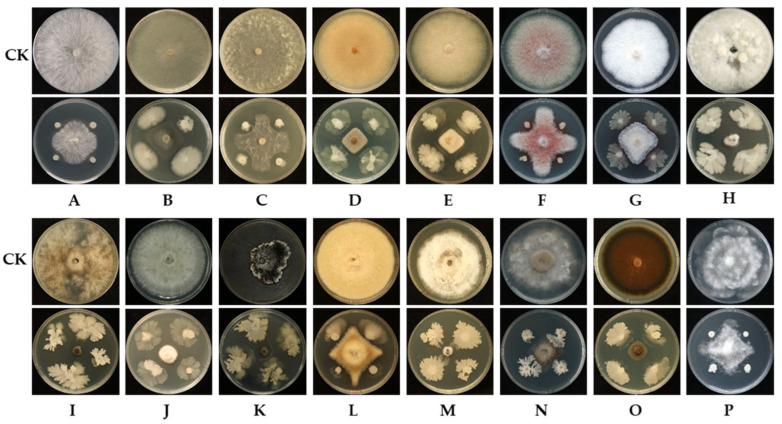
Inhibitory effects of L11-7 against fungal phytopathogens. CK: Inoculate pathogen only; (**A**): *Sclerotium rolfsii*; (**B**): *Rhizoctonia solani*; (**C**): *Sclerotinia sclerotiorum*; (**D**): *Colletotrichum gloeosporioides*; (**E**): *Colletotrichum karstii*; (**F**): *Fusarium sacchari*; (**G**): *Fusarium solani*; (**H**): *Bipolaris oryzae*; (**I**): *Exserohilum turcicum*; (**J**): *Stagonospora tainanensis*; (**K**): *Pestalotiopsis portugalica*; (**L**): *Epicoccum sorghinum*; (**M**): *Pyricularia oryzae*; (**N**): *Botrytis cinerea*; (**O**): *Alternaria brassicicola*; (**P**): *Pythium ultimum*.

Strain L11-7 demonstrated diverse antagonistic effects on the growth of 16 tested plant pathogenic fungi (Figure 2). Its strongest inhibitory effect was against *Exserohilum turcicum* of corn, with an inhibition rate of 89.49%, while its weakest effect was on *F. sacchari* of sugarcane, with an inhibitory rate of 56.74% (Table S5). This result indicated that strain L11-7 possesses broad-spectrum antifungal activity, suggesting that it has potential for biocontrol applications for multiple plant fungal diseases.

### 3.3. Biocontrol and PGP Traits of L11-7 In Vitro

**Figure 3:**
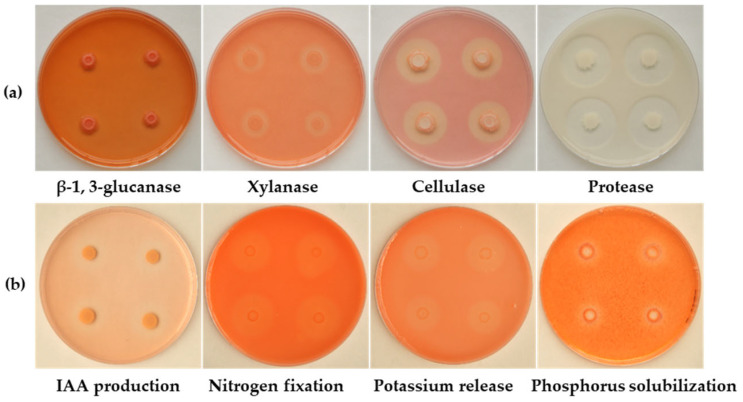
Biocontrol and PGP Traits of L11-7 In Vitro (**a**) Biological control features of L11-7 (OD_600_ = 0.6). (**b**) Plant growth-promoting properties of L11-7. The bacterium was cultured at 28 °C for 2 days (*n* = 3).

The biocontrol and PGP traits of L11-7 were investigated in vitro. Strain L11-7 tested positive for the synthesis of protease, cellulase, xylanase, and β-1,3-glucanase (Figure 3a). Furthermore, this strain could also produce IAA, fix nitrogen, release potassium, and solubilize phosphorus (Figure 3b).

### 3.4. Identification of L11-7

#### 3.4.1. Morphological Characteristics of L11-7

**Figure 4:**
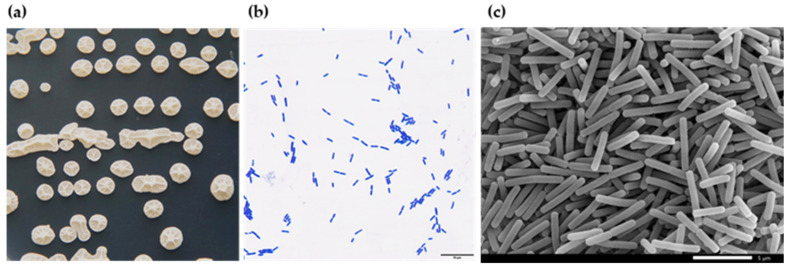
Morphological characteristics of L11-7. (**a**) L11-7 colony morphology on an LBA plate. (**b**) L11-7 is Gram-positive (scale bar = 10 μm). (**c**) Scanning electron micrograph showing the surface morphology of L11-7 (scale bar = 5 μm).

After 2 days of incubation on LB solid medium, the colonies of L11-7 were round or suborbicular, appearing creamy white to pale yellow, with a sticky, dull, and wrinkled surface (Figure 4a). Gram staining showed a purple-blue color (Figure 4b), confirming its Gram-positive nature. Scanning electron microscopy (SEM) revealed rod-shaped cells with blunt ends, measuring 2.41–4.54 μm in length and 0.58–0.69 μm in width, with no observable capsules or flagella (Figure 4c).

#### 3.4.2. Molecular Identification of Strain L11-7

**Figure 5:**
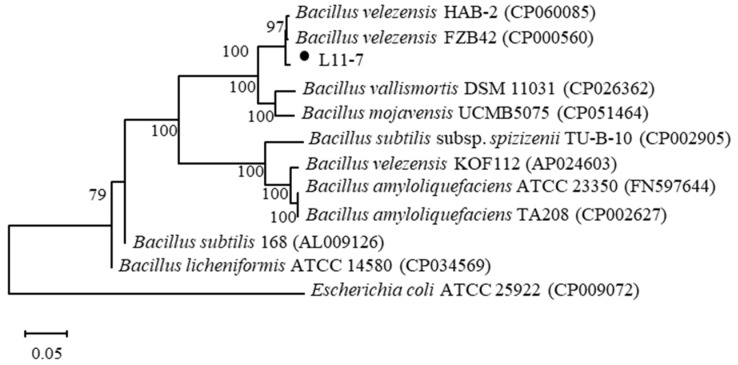
Phylogenetic tree constructed using the 16S rRNA, *gyrB*, and *rpoB* gene sequences. The numbers in parentheses refer to the GenBank accession number of the strain. The number on the branch point indicates the confidence of the branch, and the scale represents evolutionary distance.

The 16S rRNA, *gyrB* (DNA gyrase subunit B), and *rpoB* (RNA polymerase beta subunit) housekeeping genes were successfully amplified from the genomic DNA of L11-7, yielding fragments of 1511, 1259, and 580 bp, respectively. These sequences shared over 99% identity with the corresponding sequences of *B. velezensis* HAB-2 and *B. velezensis* FZB42. A phylogenetic tree constructed using the concatenated sequences of these three genes via the neighbor-joining (NJ) method showed that L11-7 clustered closely with *B. velezensis* HAB-2 and *B. velezensis* FZB42 (Figure 5). Based on these results, L11-7 was identified as *B. velezensis*. The 16S rRNA, *gyrB*, and *rpoB* gene sequences have been deposited in the GenBank database under the accession numbers PV789342, PV837614, and PV837615, respectively.

### 3.5. Genomic Properties of L11-7

**Figure 6:**
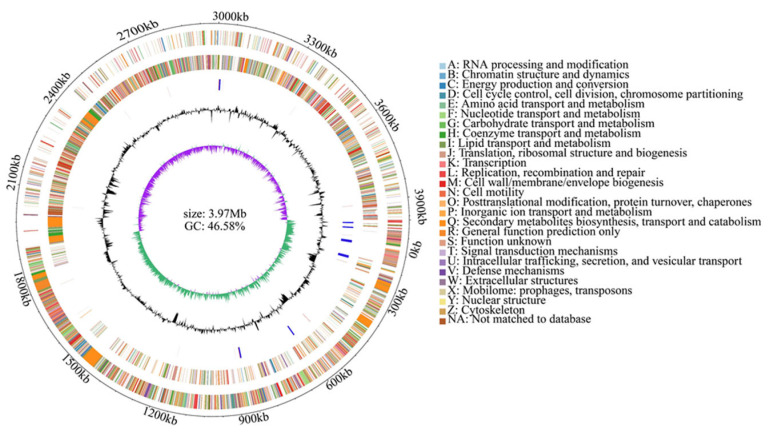
Genome map of *Bacillus velezensis* strain L11-7. From outside to inside, the first circle represents the genome coordinates; the second and third circles represent COG annotated genes on the positive and negative strands of the genome sequence, classified according to color; the fourth circle represents rRNA (blue) and tRNA (red); the fifth circle represents the average GC content curve; the sixth circle represents the GC skew curve, with green and purple representing the G/C ratio relationship.

The genome of *B. velezensis* L11-7 consists of a single circular chromosome of 3,973,740 base pairs without plasmids (Table S6). The assembled genome was deposited at the National Genomics Data Center (NGDC) under the accession number SAMC5307781. It has a total GC content of 46.58%, and contains 2795 protein-coding genes, 86 tRNAs, and 9 copies each of 23S rRNA, 16S rRNA, and 5S rRNA. Additionally, 460 pseudogenes, 10 clustered regularly interspaced short palindromic repeats (CRISPRs), 6 genomic islands (GIs), 7 prophages, 207 repeat sequences, and 13 s metabolite biosynthetic gene clusters (BGCs) were predicted (Table S6). Figure 6 displays these genomic elements of *B. velezensis* L11-7. The assembled genome of L11-7 strain has been deposited in National Genomics Data Center (accession number: SAMC5307781).

A total of 1187 genes were functionally annotated using the Gene Ontology (GO) database, with the identified processes primarily associated with integral components of the membrane (273 genes), cytoplasm (173 genes), and ATP binding (149 genes) (Figure S2). Kyoto Encyclopedia of Genes and Genomes (KEGG) analysis led to the annotation of 2914 protein-coding genes in the L11-7 genome, and these were annotated into 5 major categories—cellular processes, environmental information processing, genetic information processing, metabolism, and organismal systems—together accounting for 76.79% of the annotated genes (Figure S3). Clusters of Orthologous Groups (COG) annotation further assigned 3602 predicted genes, which were primarily concentrated in general function prediction, amino acid transport and metabolism, and transcription processes (Figure S4).

### 3.6. Comparative Genomics Analysis of L11-7

Collinearity analysis comparing the genome of *B. velezensis* L11-7 with those of five selected model strains from the NCBI database is presented in Figure S5. The results demonstrated that L11-7 exhibited the closest phylogenetic relationship with *B. velezensis* FZB42. Most genes showed direct linear correspondence with those of *B. amyloliquefaciens* ATCC 23350 and *B. subtilis* 168, although localized genomic rearrangements such as inversions, translocations, and order disruptions were observed. Overall, synteny was well-conserved, with high sequence similarity and a close phylogenetic relationship. In contrast, L11-7 displayed poor synteny with *B. licheniformis* ATCC 14580 and *B. vallismortis* DSM 11031, indicative of a more distant evolutionary relationship.

Further confirmation was obtained through Average Nucleotide Identity (ANI) and digital DNA-DNA Hybridization (dDDH) comparisons (Table S7). The ANI and dDDH values between strain L11-7 and *B. velezensis* FZB42 exceeded established species delineation thresholds (ANI > 95.00%; dDDH > 70.0%), reaching 97.54% and 80.4%, respectively. Additionally, the ANI with other strains ranged from 72.05% to 93.68%, while dDDH values ranged from 19.8% to 55.5%. These results collectively confirmed that strain L11-7 belongs to *B. velezensis*.

### 3.7. Secondary Metabolite Gene Clusters in B. velezensis L11-7

**Figure 7:**
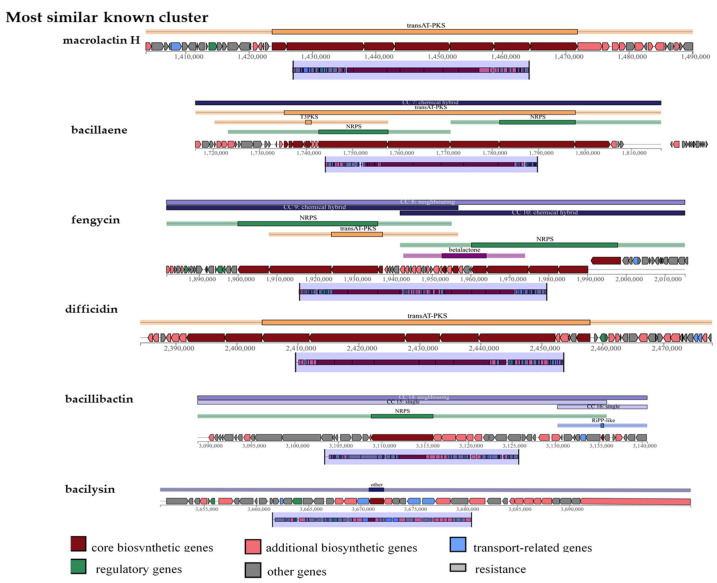
Gene cluster of secondary metabolites in the genome of *B. velezensis* L11-7.

antiSMASH prediction revealed that *B. velezensis* L11-7 encodes 13 biosynthetic gene clusters (BGCs) (Table 1). These include nonribosomal peptide synthetase (NRPS) clusters, transacyl transferase polyketide synthetase (transAT-PKS) clusters, a polyketide synthase (PKS)-like cluster, terpene clusters, a lanthipeptide-class-ii cluster, a type 3 polyketide synthetase (T3PKS) cluster, and a post-tRNA-modified peptide (RiPP-like) cluster, among other gene cluster types. Notably, six BGCs showed 100% similarity to previously reported clusters responsible for macrolactin H, bacillaene, fengycin, difficidin, bacillibactin, and bacilysin production (Table 1, Figure 7). Gene cluster 2, responsible for surfactin synthesis, showed 78% similarity with the respective previously characterized clusters. Furthermore, cluster 1 showed only 22% similarity to the rhizocticin A biosynthetic gene cluster. Interestingly, four gene clusters (4, 5, 9, and 10) in the genome showed no sequence similarity to known bacterial gene clusters, suggesting that L11-7 may harbor novel secondary metabolite synthetic pathways.

### 3.8. Genes Associated with Biological Control and Plant Growth Promotion in the L11-7 Genome

Strain L11-7 displayed significant biocontrol and PGP properties under in vitro conditions, including hydrolase activity (cellulase, glucanase, and protease production) and nitrogen and phosphorus solubilization capabilities (Figure 3). Genomic analysis further revealed that strain L11-7 harbors genes encoding various fungal cell wall-degrading enzymes, as annotated in the CAZymes database, including chitinases, α-amylases, and endoglucanases (Table 2). Genome annotation also identified multiple PGP-related gene clusters (Table 3), including those with roles in IAA biosynthesis (*trpA/B/C/D/F*), nitrogen fixation (*nifH/L/M/U*), phosphate metabolism (*phnC/E/F/R* and *pstA/B/C/S*), potassium transport (*ktrA/C/D* and *kbp*), and iron acquisition (*fbpA/B*, *fbpB,* and *fetB*).

## 4. Discussion

The biological control of plant diseases using beneficial microorganisms is a promising plant protection strategy [19]. *Bacillus* species, especially *B. velezensis*, possess multiple mechanisms for combating phytopathogens and have emerged as highly promising biocontrol agents for the management of soil-borne diseases [39]. However, the exploitation of *B. velezensis* as a biocontrol agent against stem basal rot in passion fruit remains largely unexplored. In this study, we identified *B. velezensis* strain L11-7 as an antagonist of *F. solani* and subsequently demonstrated its biocontrol potential. Meanwhile, genomic analysis was performed to determine the mechanisms underlying its biocontrol activity and PGP traits.

Many *Bacillus* species produce a variety of hydrolases, such as chitinase, pectinase, cellulase, β-1,3-glucanase, and protease, all of which are crucial for breaking down fungal pathogen components [40]. Chitinases, for instance, inhibit fungal growth by degrading chitin and disrupting cell wall integrity [41]. Pectinase, protease, and cellulase similarly degrade the cell walls and membranes of phytopathogens. Additionally, cellulases and glucanases can work synergistically with chitinases, significantly boosting their antagonistic effect [42]. Xylanase, a key enzyme responsible for the conversion of lignocellulose into fermentable sugars, has also been linked to the biocontrol activity of some *Bacillus* species [42]. These hydrolases also contribute to the indirect antagonistic effects exerted by plants in defense and growth promotion. In this study, we found that *B. velezensis* L11-7 can produce protease, cellulase, β-1,3-glucanase, and xylanase, consistent with the results of Huang et al. (2023) [43]. In the subsequent genomic analysis, CAZy functional annotation results indicated that L11-7 possesses multiple genes, such as *bcsB*, *lpmO*, and *xynC*, that encode these antifungal hydrolases. Future research should focus on optimizing cultivation conditions to maximize hydrolase activity, thereby clarifying their role in the biocontrol and plant growth-promoting effects of L11-7.

Numerous biocontrol microorganisms directly inhibit or kill pathogens by synthesizing antagonistic secondary metabolites and releasing antimicrobial volatile organic compounds [44]. A key antagonistic mechanism employed by *B. velezensis* involves the production of lipopeptides and polyketide antibiotics such as surfactin, fengycin, macrolactin, bacillaene, difficidin, bacillibactin, and bacilysin [20,45,46,47]. Surfactin impacts biofilm formation and colonization, and can also resist pathogenic fungi, mycoplasma, and viruses [48,49]. Furthermore, surfactin has also been shown to induce apoptosis-like cell death and disrupt energy metabolism in *F. solani*, while simultaneously reducing mitochondrial membrane potential [14]. Fengycin disrupts fungal cell membrane integrity and causes cell death. A recent study attributed *B. velezensis’s* growth inhibitory effect against *F. solani* to the presence of fengycin (named BVAP) [15]. Polyketides (bacillaene, difficidin, and macrolactin) and bacilysin, a dipeptide antibiotic, are recognized for their antibacterial activities [47], with bacillaene also showing antifungal properties [50]. Bacilysin produced by *B. velezensis* strain FZB42 is responsible for inhibiting *Phytophthora sojae*, an oomycete pathogen [51]. Our genome sequencing of *B. velezensis* L11-7 revealed the presence of NRPS and PKS gene clusters, consistent with the strong antifungal activity of *B. velezensis* L11-7 strain. Moreover, antimicrobial compounds, such as surfactin and fengycin, not only directly suppress the growth of pathogens but also induce systemic resistance in host organisms [52,53]. In this study, the L11-7 strain exhibited a lower inhibition rate than other strains (L11-3, L11-6, L13-1, L19-3, and M12-5) in the dual-culture plate assay but demonstrated the highest control efficacy in pot experiments. The inhibitory effect in plate assays depends on antagonistic substances produced by the microorganisms and the biomass of target fungi, whereas the control effect in pot or field experiments is influenced by multiple factors including pathogens, plants, antagonists, and environment environmental conditions [54,55]. Given that L11-7 harbors NRPS gene clusters associated with surfactin and fengycin biosynthesis, we hypothesize that this strain may induce resistance in passion fruit plants, thereby enhancing its biocontrol performance. While various *B. velezensis* strains produce iturin [47], we did not detect a gene cluster encoding this lipopeptide in strain L11-7.

Rhizocticin A is an antifungal phosphono-oligopeptide first reported in *B. subtilis* ATCC6633, with known inhibitory activity against both fungi and nematodes [47,56]. To date, only a few *Bacillus* species, such as *B. subtilis* and *B. halotolerans*, have been reported to produce rhizoctin A [57]. Whole-genome sequencing annotation of strain L11-7 revealed the presence of a gene cluster associated with rhizocticin A biosynthesis. However, the antibiotic potential of rhizocticin A in *B. velezensis* has not yet been experimentally validated. Further research, including gene knockout experiments and gene expression profiling, is needed to determine which antagonistic compounds are produced by strain L11-7 and how they contribute to biocontrol activity.

PGP attributes have been reported in a variety of strains of *B. velezensis*. They can promote plant growth through multiple mechanisms, such as nitrogen fixation, phosphate solubilization, phytohormone and siderophore production, and the induction of systemic disease resistance [22]. In the present study, we found that the L11-7 strain possesses plant growth-promoting properties, characterized by the ability to produce IAA, fix nitrogen, solubilize phosphate, and release potassium (Figure 3). Moreover, the genome of this strain harbors several genes potentially related to plant growth promotion. A complete tryptophan biosynthetic pathway (*trpA*, *trpB*, *trpC*, *trpD*, and *trpF*), linked to IAA production, was identified, providing genetic-level evidence that L11-7 is capable of synthesizing IAA. Furthermore, L11-7 harbors key nitrogen fixation-related genes, including *nifL*, *nifM*, *nifU*, and *nifH*, which are primarily involved in the synthesis, assembly, regulation, and electron transfer processes of nitrogenase. Its genome also encodes phosphate transporter genes (*phnC* and *phnE*) and transcriptional regulators (*phnF* and *phnR*) associated with phosphate metabolism, facilitating efficient phosphorus uptake and utilization. Regarding potassium transport, members of the *ktr* and *kbp* gene families, which are closely associated with K^+^ uptake and transport, are also present in L11-7, further supporting its role in enhancing plant potassium availability. Finally, the genome of L11-7 also contained several genes or gene clusters potentially related to siderophore production. Siderophores exhibit a strong affinity for Fe^3+^, and compete with plant pathogenic bacteria and fungi for available iron, thereby suppressing their growth and inhibiting their ability to cause plant diseases [20]. Siderophores also increase nutrient availability, which promotes plant growth and development [58]. Future work should focus on determining whether strain L11-7 produces siderophores and their putative relationship with its biocontrol characteristics. In summary, these findings indicate that L11-7 has the potential to promote plant growth through several mechanisms, such as the production of IAA and siderophores, nitrogen fixation, and phosphate solubilization. Various Bacillus velezensis strains, including FZB42 and GB03, have been widely used in agriculture due to their antifungal activity and plant growth-promoting properties [59,60]. Therefore, *B. velezensis* L11-7 is likely to prevent passion fruit stem basal rot through multiple mechanisms, including producing antagonistic substances, inducing systemic resistance, and regulating passion fruit growth and stress responses. These findings highlight its potential as a biocontrol agent.

This study demonstrated the biocontrol potential of *B. velezensis* L11-7 against *F. solani*, along with its broad-spectrum antimicrobial activity. L11-7 can produce a range of bioactive metabolites with both antimicrobial and PGP properties. Genome analysis revealed that *B. velezensis* L11-7 harbors metabolic pathways responsible for producing diverse secondary metabolites with multiple functions, including plant pathogen inhibition, cell wall degradation, and plant growth promotion, providing molecular insights into its biocontrol and PGP mechanisms. Future research should aim to optimize the fermentation conditions for this strain, purify and characterize its bioactive compounds, and systematically evaluate the field application efficacy of both the strain and its metabolites. Additionally, further investigation into the mechanisms behind the interactions among the strain, plants, and the environment is warranted to comprehensively assess its practical applicability and potential for large-scale implementation.

## 5. Conclusions

*Bacillus velezensis* strain L11-7 exhibited strong antagonistic effects against *F. solani*, demonstrated broad-spectrum antagonistic activity against multiple phytopathogenic fungi, and exhibited several plant growth-promoting traits. The genome of L11-7 consists of a 3.97 Mb circular chromosome harboring 13 s metabolite biosynthetic gene clusters, genes encoding essential cell wall hydrolases, and several genes related to plant growth promotion. Collectively, these findings suggest that *B. velezensis* strain L11-7 exhibits both biocontrol potential and plant growth-promoting properties. Accordingly, it represents a promising biocontrol candidate for managing passion fruit stem basal rot. Additionally, it may serve as a valuable microbial resource for the development of biofungicides and plant growth stimulants, providing both theoretical and practical foundations for sustainable agriculture.

## Figures and Tables

**Table 1:** Secondary metabolite biosynthetic gene clusters in *Bacillus velezensis* L11-7.

ID	Gene Cluster Type	Compound	Similarity (%)	Size (Kb)
Cluster 1	NRPS, transAT-PKS	rhizocticin A	22	69.9
Cluster 2	NRPS	surfactin	78	64.8
Cluster 3	PKS-like	butirosin A/butirosin B	7	41.2
Cluster 4	Terpene	–	–	–
Cluster 5	Lanthipeptide-class-II	–	–	–
Cluster 6	TransAT-PKS	macrolactin H	100	86.4
Cluster 7	TransAT-PKS, T3PKS, NRPS	bacillaene	100	100.2
Cluster 8	NRPS, transAT-PKS, betalactone	fengycin	100	133.5
Cluster 9	Terpene	–	–	–
Cluster 10	T3PKS	–	–	–
Cluster 11	TransAT-PKS	difficidin	100	93.8
Cluster 12	NRPS, RiPP-like	bacillibactin	100	51.8
Cluster 13	Other	bacilysin	100	41.4

Note: “–“ indicates unknown.

**Table 2:** Genes associated with hydrolytic enzymes in the L11-7 genome.

Hydrolase	Gene	Gene Annotation	Locus Tag
Amylase	*malS*	Alpha-amylase	322,259–324,238
Cellulase	*bcsB*	Cellulose synthase operon protein B	463,627–465,741
	–	Glycosyl hydrolase family 5	1,892,248–1,893,747
	*lpmO*	Lytic polysaccharide mono-oxygenase, cellulose-degrading	1,849,287–1,849,907
Glucanase	*bcsZ*	Endoglucanase	458,744–459,829
	*–*	Arabinogalactan endo-1,4-beta-galactosidase	1,181,138–1,182,250
	*–*	Beta-glucanase, Glycosyl hydrolases family 16	3,793,329–3,794,060
Xylanase	*xynC*	Glucuronoarabinoxylan endo-1,4-beta-xylanase	1,896,831–1,898,102
	*xynA*	Endo-1,4-beta-xylanase	3,579,625–3,580,266
Chitinase	*–*	Spore cortex-lytic enzyme	2,316,236–2,317,105
	*–*	Peptidoglycan/xylan/chitin deacetylase	3,579,625–3,580,266
	*–*	Glycosyl hydrolases family 18	24,670–25,950
Pectinase	*–*	Pectin lyase	3,803,238–3,804,302

Note: “–” indicates that the gene name is unknown.

**Table 3:** Genes predicted to be associated with plant growth-promoting (PGP) activities in the L11-7 genome.

Pgp Activities	Gene	Gene Annotation	locus Tag
IAA production	*trpA*	Tryptophan synthase alpha chain	2,289,716–2,290,513
	*trpB*	Tryptophan synthase beta chain	2,290,506–2,291,708
	*trpC*	Indole-3-glycerol phosphate synthase	2,292,347–2,293,099
	*trpD*	Anthranilate phosphoribosyltransferase	3,823,136–3,824,437
	*trpF*	Phosphoribosyl anthranilate isomerase	2,291,689–2,292,342
Nitrogen fixation	*nifL*	Nitrogen fixation negative regulator	1,894,579–1,895,979
	*nifM*	Nitrogen fixation protein	989,099–989,950
	*nifU*	Nitrogen fixation protein and related proteins	3,182,807–3,183,250
	*nifH*	4Fe-4S iron sulfur cluster binding proteins	1,657,745–1,658,638
	*sufU*	Iron-sulfur cluster assembly scaffold protein	3,182,807–3,183,250
Phosphate metabolism	*phnC*	Phosphonate ABC transporter ATP-binding protein	386,204–386,947
	*phnF*	Phosphonate metabolism transcriptional regulator	386,204–386,947
	*PhnE*	Phosphonate ABC transporter, permease protein	3,308,485–3,309,138
	*phnR*	Phosphonate utilization transcriptional regulator	778,830–779,546
	*pstA*	Phosphate transport system permease protein	2,559,909–2,560,793
	*pstB*	Phosphate transport system ATP-binding protein	2,558,290–2,559,072
	*pstC*	Phosphate transport system permease protein	2,560,793–2,561,722
	*pstS*	Phosphate transport system substrate-binding protein	2,561,771–2,562,673
	*phoH*	Phosphate starvation-inducible protein and related proteins	2,594,378–2,595,337
Potassium	*kbp*	Cytoplasmic potassium-binding protein	1,254,360–1,255,019
	*trkA*	Voltage-gated potassium channel	3,047,898–3,048,884
	*ktrC*	System potassium transporter	1,415,854–1,416,519
	*ktrD*	System potassium uptake protein	1,323,727–1,325,079
Siderophore	*fbpA*	Fur-regulated basic protein A	489,207–489,386
	*fbpB*	Fur-regulated basic protein B	489,074–489,220
	*feoB*	Ferrous iron transport protein B	1,623,634–1,624,482
	*–*	Iron-hydroxamate ABC transporter substrate-binding protein	3,830,918–3,831,880
	*–*	ABC-type Iron 3+-hydroxamate transport system	3,243,827–3,244,765
	*fetB*	Iron transport system permease protein	1,201,897–1,202,658

Note: “–” indicates that the gene name is unknown.

## Data Availability

The original contributions presented in this study are included in the article/Supplementary Material. Further inquiries can be directed to the corresponding author.

## Supplementary Materials

The following supporting information can be downloaded at: https://www.mdpi.com/article/10.3390/microorganisms13092084/s1, Figure S1: Inhibition effects of different bacterial strains on the growth of *Fusarium solani*; Figure S2: The horizontal axis represents the content of each category in GO, and the vertical axis represents the number of genes; Figure S3: The horizontal coordinate represents the number of annotated genes under the Pathway classification; Figure S4: The horizontal axis represents the classification contents of COG, and the vertical axis represents the number of genes; Figure S5: Collinearity analysis of *Bacillus velezensis* L11-7. Table S1: The *Bacillus* and phytopathogenic fungal strains used in this study; Table S2: Primers and reaction procedures for housekeeping gene amplification; Table S3: Inhibitory effect of antagonistic *Bacillus* strains on the mycelium growth of *Fusarium solani*; Table S4: Effect of different treatments on stem base rot of passion fruit; Table S5: Inhibition rate of strain L11-7 on 16 tested plant pathogenic fungi; Table S6: Genome features of strain L11-7; Table S7: Average Nucleotide Identity (ANI)- and digital DNA-DNA Hybridization (dDDH of *Bacillus velezensis* strain L11-7 and its relatives.
