# Supplementary material for: Identification and Genomic Insights into the Biological Control and Growth-Promoting Mechanism of Bacillus velezensis L11-7, a Potential Biocontrol Agent of Passion Fruit Stem Basal Rot
Source: Microorganisms. 2025 Sep 7;13(9):2084. doi: 10.3390/microorganisms13092084 (PMC12472520; doi:10.3390/microorganisms13092084)
Supplement: Supplementary file 1 [file microorganisms-13-02084-s001.zip › microorganisms-3848662-supplementary.pdf]

Supplementary Materials

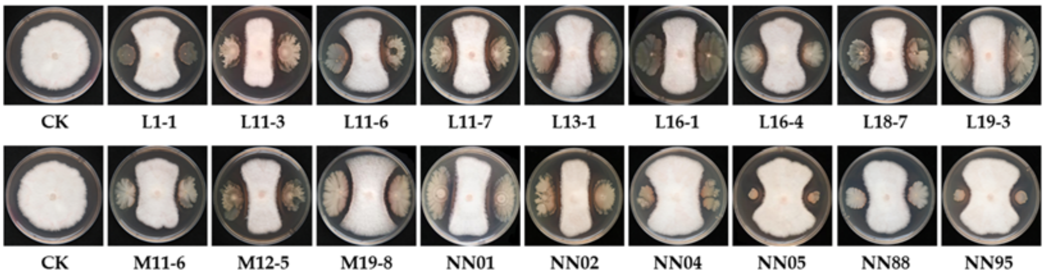

Figure S1. Inhibition effects of different bacterial strains on the growth of *Fusarium solani*. CK: the control group inoculated only with *F. solani*.

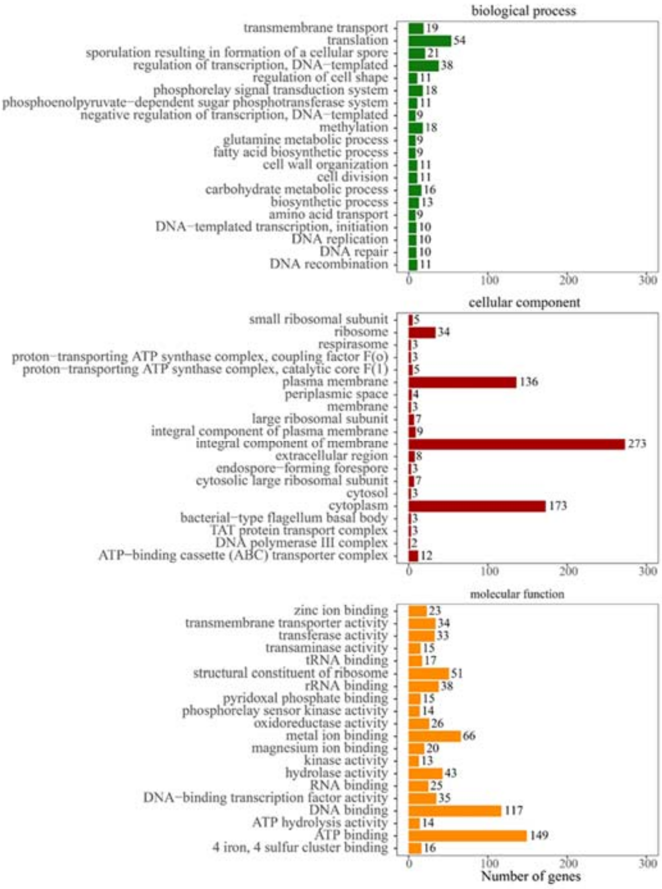

Figure S2. The horizontal axis represents the content of each category in GO, and the vertical axis represents the number of genes. This figure shows the gene enrichment of each secondary function of GO in the entire genetic context, reflecting the status of each secondary function in this context.

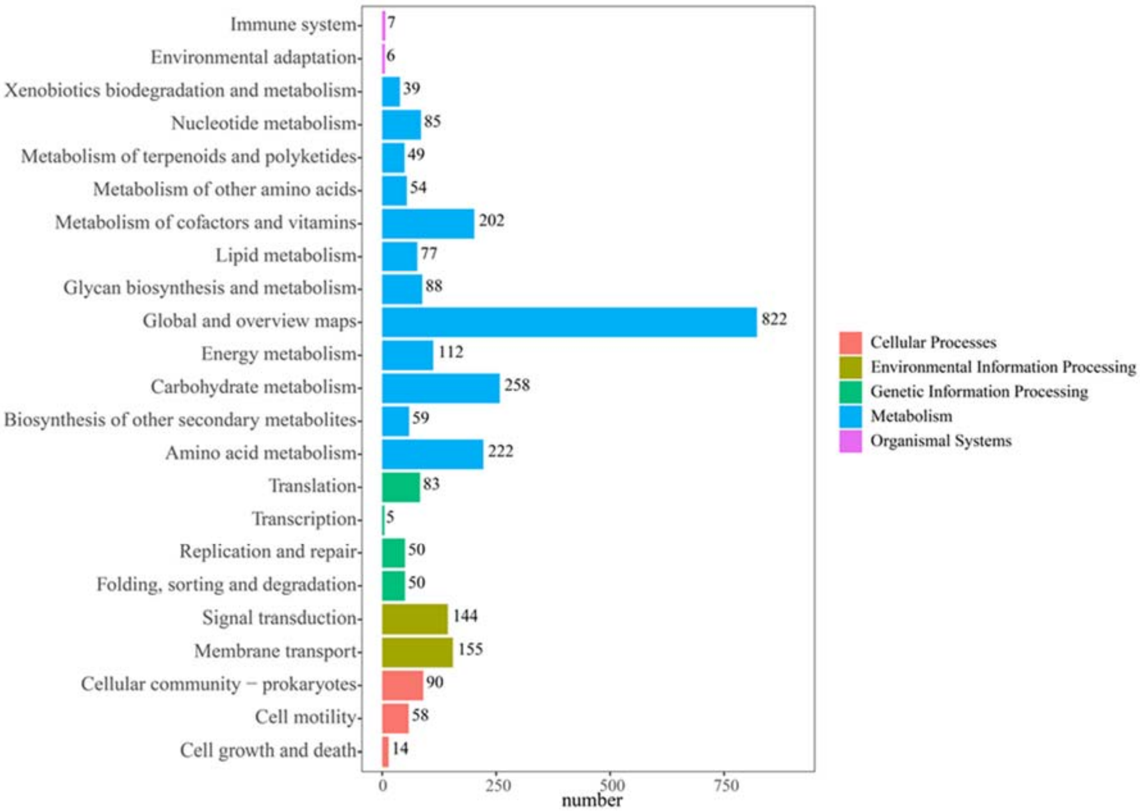

Figure S3. The horizontal coordinate represents the number of annotated genes under the Pathway classification. The vertical coordinate represents the Pathway classification, and different colors indicate the different major categories to which it belongs.

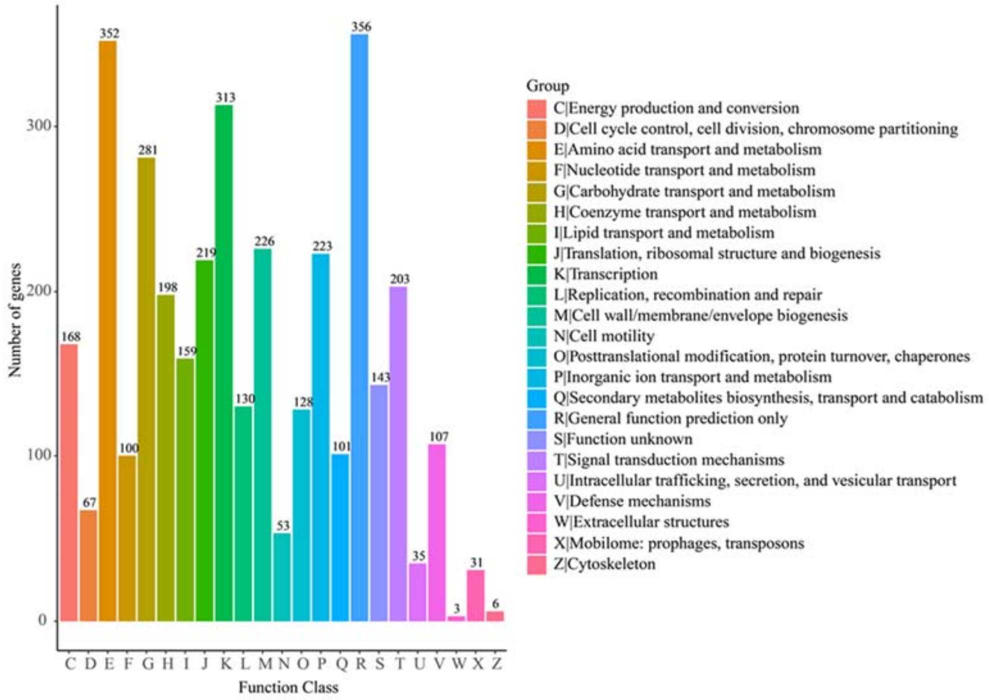

Figure S4. The horizontal axis represents the classification contents of COG, and the vertical axis represents the number of genes.

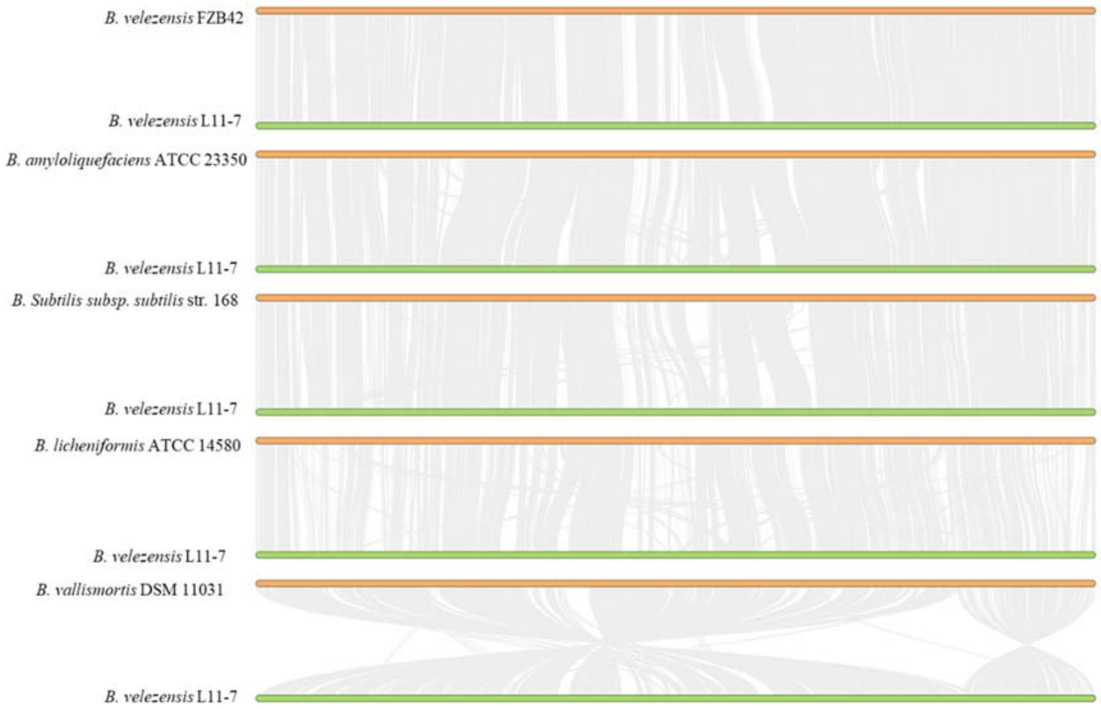

Figure S5. Collinearity analysis of *Bacillus velezensis* L11-7. Analysis of covariance between strain L11-7 and *B. velezensis* FZB42, *B. amyloliquefaciens* ATCC 23350, *B. subtilis* 168, *B. licheniformis* ATCC 14580, and *B. vallismortis* DSM 11031, respectively. The top axis represents the reference genome coordinates; the middle and bottom axes display the sequenced genome of L11-7.

Table S1. The *Bacillus* and phytopathogenic fungal strains used in this study

	Strain	Source/Host plant
Bacterial strains	M5-7	Zucchini rhizosphere soil
	M6-2	Mustard greens rhizosphere soil
	M10-4, M10-7, M10-8, M10-9	Variegated agave rhizosphere soil
	M11-6, M11-8	Monstera deliciosa rhizosphere soil
	M12-2, M12-3, M12-5	Chinese hibiscus rhizosphere soil
	M19-5, M19-6, M19-8	Potato rhizosphere soil
	M20-3, M20-12, M20-10, M21-16, L14-3	Tobacco rhizosphere soil
	L1-1, L1-7	Lettuce rhizosphere soil
	L4-4	Litchi rhizosphere soil
	L10-5	Peanut rhizosphere soil
	L11-3, L11-4, L11-6, L11-7	Pitaya rhizosphere soil
	L13-1	Chinese chive rhizosphere soil
	L15-5, L16-1, L16-4, L17-3, L18-7, L19-3, L19-5, L19-6、L19-7	Cassava rhizosphere soil
	NN01, NN04	Mulberry stem
	NN02	Mulberry branch
	NN05, NN88, NN95	Mulberry rhizosphere soil
Phytopathogenic fungi	*Sclerotium rolfsii*	Peanut
	Rhizoctonia solani	Rice
	Sclerotinia sclerotiorum	Lettuce
	Colletotrichum gloeosporioides	Citrus
	Colletotrichum karstii	Passion fruit
	Fusarium sacchari	Sugarcane
	Fusarium solani	Passion fruit
	Bipolaris oryzae	Rice
	Exserohilum turcicum	Corn
	Stagonospora tainanensis	Sugarcane
	Pestalotiopsis portugalica	Oil-tea camellia
	Epicoccum sorghinum	Sugarcane
	Pyricularia oryzae	Rice
	Botrytis cinerea	Tomaro
	Alternaria brassicicola	Chinese cabbage
	Pythium ultimum	Cucumber

Table S2. Primers and reaction procedures for housekeeping gene amplification.

Gene	Primer	Primer sequence	Reaction procedure
16S rRNA	27F	AGAGTTTGATCCTGGCTCAG	95℃ for 5 min, 33 cycles of 94℃ for 30 s, 55℃ for 30 s, 72℃ for 45 s, final extension for 72℃ 5 min
	1492R	TACGGCTACCTTGTTACGACTT	
gyrB	UP1	GAAGTCATCATGACCGTTCTGCAYGCNGGNGGNAARTTYGA	
	UP2r	AGCAGGGTACGGATGTGCGAGCCRTCNACRTCNGCRTCNGTCAT	
rpoB	rpoB-F	AGGTCAACTAGTTCAGTATGGAC	
	rpoB-R	AAGAACCATAACCGGCAACTT	

Table S3. Inhibitory effect of antagonistic *Bacillus* strains on the mycelium growth of *Fusarium solani*

Strain	Mycelium growth inhibition rate（%）
L1-1	49.26 ±3 .43
L11-3	62.06 ± 2.56
L11-6	62.06 ± 2.56
**L11-7**	**60.09 ± 2.70**
L13-1	61.08 ± 2.63
L16-1	59.11 ± 2.76
L16-4	56.15 ± 2.96
L18-7	57.63 ± 2.86
L19-3	60.89 ± 2.63
M11-6	52.21 ± 3.23
M12-5	61.57 ±2 .60
M19-8	48.76 ± 3.46
NN01	51.98 ± 3.23
NN02	50.99 ± 3.30
NN04	54.45 ± 3.06
NN05	47.02 ± 3.56
NN88	53.46 ± 3.13
NN95	44.05 ± 3.76

Note: The values are the means ±standard errors (n=3).

Table S4. Effect of different treatments on stem base rot of passion fruit.

Treatment	Lesion mean（cm^2）	Control efficiency (%)
CK	1.97	-
L11-3	0.73	63.01±1.10
L11-6	1.80	8.38± 1.20
**L11-7**	**0.14**	**92.85± 0.20**
L13-1	0.75	61.99± 1.60
L19-3	1.65	16.45±0.71
M12-5	0.52	73.59±1.29

Note: CK: Water treatment, the values are the means (n=15), "-" indicates no calculation.

Table S5. Inhibition rate of strain L11-7 on 16 tested plant pathogenic fungi.

Plant pathogenic fungi	Colony diameter (cm)	Inhibition rate (%)
Sclerotium rolfsii	3.63 ± 0.15	57.75 ± 1.45 e
Rhizoctonia solani	3.46 ± 0.11	59.21 ± 1.10 e
Sclerotinia sclerotiorum	1.76 ± 0.05	77.91 ± 0.58 b
Colletotrichum gloeosporioides	2.10 ± 0.10	73.44 ± 1.10 cd
Colletotrichum. karstii	1.83 ± 0.05	77.08 ± 0.58 c
Fusarium sacchari	3.63 ± 0.11	56.74 ± 1.12 f
Fusarium solani	3.10 ± 0.20	57.53 ± 2.23 f
Bipolaris oryzae	1.70 ± 0.10	80.00 ± 0.96 b
Exserohilum turcicum	0.90 ± 0.10	89.49 ± 0.95 a
Stagonospora. tainanensis	1.73 ± 0.05	79.20 ± 0.56 b
Pestalotiopsis portugalica	0.96 ± 0.11	81.04 ± 1.84 b
Epicoccum sorghinum	3.33 ± 0.11	60.78 ± 1.10 e
Pyricularia. oryzae	0.96 ± 0.05	86.81 ± 0.64 a
Botrytis cinerea	2.46 ± 0.28	70.98 ± 2.77 cd
Alternaria brassicicola	1.76 ± 0.05	78.09 ± 0.58 b
Pythium ultimum	3.46 ± 0.11	59.21 ± 1.10 e

Note: The data presented in the table represent the means ± standard errors (n=3). Different letters on each number define groups of treatments that showed significant differences at the $P < 0.05$ by one-way analysis of variance (ANOVA) and Tukey test.

Table S6. Genome features of strain L11-7.

Feature	Chromosome characteristics
Genome topology	Circular
Chromosome size (bp)	3,973,740
GC content	46.58%
CDS	3,795
Gene average length (bp)	897
tRNA	86
23S RNA	9
16S RNA	9
5S RNA	9
pseudogene	460
CRISPR	10
GIs	6
repeat sequences	207
BGCs	13
Genes assigned to GO	1187
Genes assigned to KEGG	2914
Genes assigned to COG	3602

Table S7. Average Nucleotide Identity (ANI)- and digital DNA-DNA Hybridization (dDDH of *Bacillus velezensis* strain L11-7 and its relatives.

Strain	ANI (%)	dDDH (%)
B. velezensis FZB42	97.54	80.4
B. amyloliquefaciens ATCC 23350	93.68	55.5
B. subtilis 168	76.33	20.7
B. vallismortis DSM 11031	76.41	20.6
B. licheniformis ATCC 14580	72.05	19.8
